# New Promising Therapeutic Approach for Refractory Corneal Epithelial Defects

**DOI:** 10.7759/cureus.39324

**Published:** 2023-05-22

**Authors:** Rodrigo Vilares Morgado, Rodolfo Moura, Raúl Moreira, Fernando Falcão-Reis, João Pinheiro-Costa

**Affiliations:** 1 Department of Ophthalmology, São João Universitary Hospital Center, Porto, PRT

**Keywords:** cornea and external eye diseases, scleral contact lens, topical autologous serum, corneal wound healing, corneal epithelial defect, corneal epithelium

## Abstract

The purpose of this case report is to describe a case of continuous wear of a gas-permeable mini-scleral contact lens with a fluid reservoir of autologous serum (AS) combined with AS drops as a successful empirical and accessible alternative therapeutic option for refractory persistent epithelial defects in a patient with severe neurotrophic keratopathy (NK) due to severe dry eye disease and chronic contact lens wear. A 61-year-old Caucasian female with bilateral NK presented a history of multiple episodes of bilateral persistent epithelial defects, having already been submitted to three tectonic-penetrating keratoplasties in her left eye (OS). In May 2017, the patient developed *de novo* refractory central neurotrophic ulcers in both eyes (OU), unresponsive to conventional treatment with preservative-free lubricants, topical antibiotics, topical anti-inflammatory agents, and oral doxycycline. By March 2018, after initiating hourly AS eyedrops, the ulcer in her right eye (OD) improved to a smaller ulcer, while her OS presented complete graft re-epithelialization. In May 2018, her OD neurotrophic ulcer was complicated with fungal and subsequent bacterial secondary infection. Eventually, a therapeutic penetrant keratoplasty was required for her OD. Subsequently, her OD graft developed a *de novo* 6x6mm central persistent epithelial defect unresponsive to all the aforementioned therapeutic strategies. After months of unsuccessful treatment, a new therapeutic option was experimented with: a gas-permeable mini-scleral contact lens in combination with AS eyedrops. After two weeks of this treatment regimen, the corneal epithelium eventually started to regenerate, and four weeks later, the cornea was completely re-epithelized. To date, there are no signs of recurrence of the corneal epithelial defect/ulcer.

## Introduction

A healthy corneal epithelium consists of multilayered squamous cells with self-renewing ability. In any corneal disease, re-epithelialization is of great importance, as corneal thinning seldom progresses if the epithelium is intact [1]. Persistent corneal epithelial defects refer to the loss of corneal surface integrity, which is not able to heal within the usual two-week time span, generally in the setting of significant ocular surface disorders, such as severe dry eye disease or neurotrophic keratopathy (NK) [2, 3].

NK develops when there is loss of corneal trigeminal innervation, resulting in reduced corneal sensation, with inadequate tear production and loss of the protective sensory stimulus. Corneal sensory nerves serve nociceptor and trophic functions, which can be affected independently or simultaneously. Loss of trophic function results in intracellular edema and epithelial breakdown and exfoliation, thus exposing the corneal stroma and making it susceptible to enzymatic degradation [4]. NK is characterized by corneal hypoesthesia or anesthesia and, thus, often by a discrepancy between the clinical picture and the patient's symptoms. Therefore, the diagnosis of NK is often challenging [5]. NK is graded according to the severity of the clinical signs (Mackie classification 1995) [4]. In stage one, mild NK, there is an epithelial irregularity with mild opacification and small focal epithelial defects. In stage two, moderate NK, larger persistent epithelial defects develop, usually with rolled and thickened edges, assuming a punched-out configuration with underlying stromal opacification. In stage three, severe NK, stromal melting occurs, and secondary infection or corneal perforation may subsequently develop. Both moderate and severe NK can profoundly affect vision and adversely impact the quality of life [4]. Bian et al. recently conducted a retrospective analysis of visits associated with a diagnosis of NK between 2013 and 2018 using the American Academy of Ophthalmology Intelligent Research in Sight (IRIS®) Registry, including 31,915 eyes from 27,483 patients with a diagnosis of NK. In their study, the mean age at initial diagnosis of NK was 68.0 ± 16.0 years, with the majority of cases being unilateral at presentation (58.14%; bilateral in 16.13%), while the most frequent concomitant diagnoses included herpetic keratitis (33.70%), diabetes (31.59%), and corneal dystrophies (14.28%) [6].

Treating persistent epithelial defects secondary to NK can be a true medical challenge as they are frequently resistant to conventional therapies. The most common treatment methods include conservative treatment, such as preservative-free tear substitutes, topical antibiotics, anti-collagenase agents, and topical or systemic anti-inflammatory agents [7]. Further surgical and non-surgical treatment modalities have been developed: punctal occlusion, nerve growth factor (NGF) eyedrops, autologous serum (AS) eye drops, amniotic membrane transplantation (AMT), and corneal neurotization from the supratrochlear nerve using a sural nerve graft. Additionally, protection of the ocular surface with temporary or permanent tarsorrhaphy, botulinum-toxin-induced ptosis, and therapeutic scleral contact lenses have also been used as therapeutic options for these patients.

In our ophthalmology department, we have used all these aforementioned conventional and more recent therapies for patients with NK. However, recently, we encountered a particularly challenging case that required a new therapeutic approach. Thus, the purpose of this case report is to describe a case of continuous wear of a gas-permeable mini-scleral contact lens with a fluid reservoir of AS combined with AS drops as a successful empirical and accessible alternative therapeutic option for refractory persistent epithelial defects in a patient with severe NK.

## Case presentation

A 61-year-old Caucasian female with a history of chronic contact lens wear, severe dry eye disease, and bilateral NK had been followed in our cornea clinic since 2002. The patient had a personal history of type 2 diabetes mellitus, diagnosed when the patient was 50 years old, with poor control in the first five years of the disease but with regular glycated hemoglobin levels under 7.5% in the latter five years, and systemic hypertension. Regular medications included oral antidiabetic and antihypertensive drugs. Regarding her ophthalmological history, the patient presented a history of multiple episodes of bilateral persistent epithelial defects, having already been submitted to three tectonic penetrating keratoplasties in her left eye (OS), in 2003, 2010, and 2016, due to successive neurotrophic corneal ulcers with corneal melting and eventual perforation. The patient had also undergone uncomplicated cataract surgery in both eyes (OU) in 2010. Secondary autoimmune causes for her severe dry eye disease had previously been ruled out.

**Figure 1:**
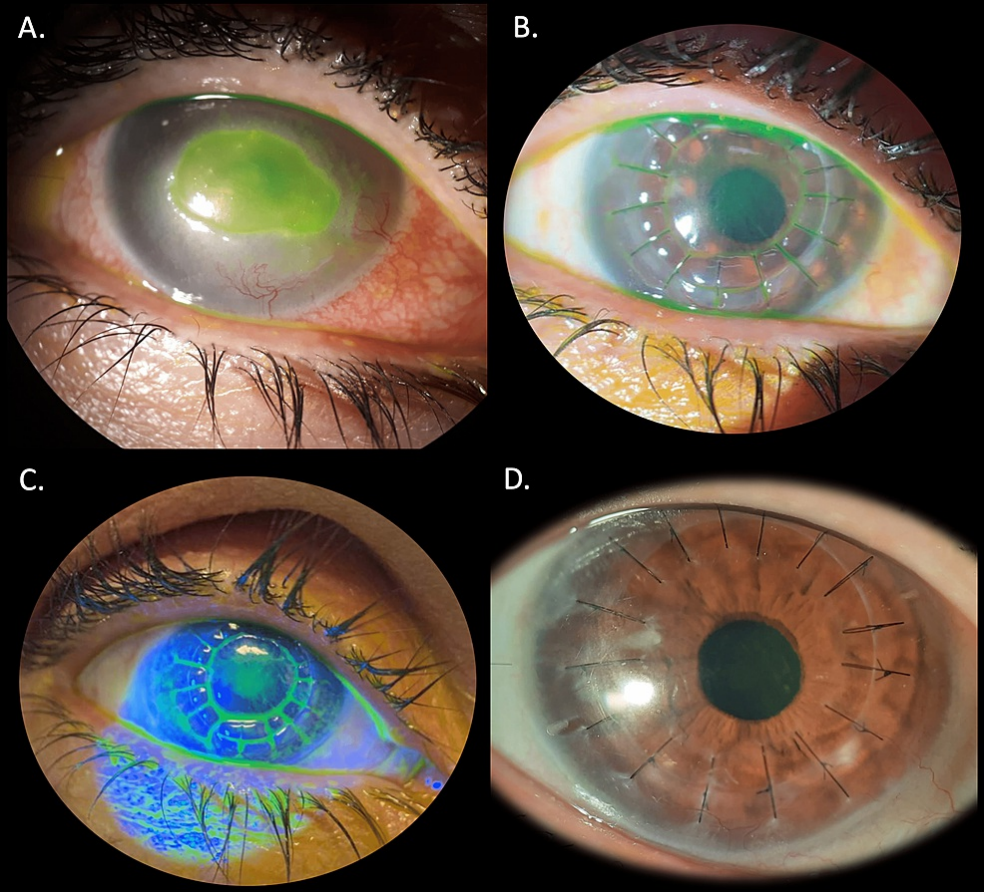
Evolution over time of the OD anterior segment (A) September 2018 slit-lamp photography showing the extensive and central neurotrophic ulcer with stromal melting and haze in OD. (B, C) Slit-lamp examination after four weeks of scleral lens + autologous serum usage, revealing a transparent and fully epithelized corneal graft. (D) February 2021 OD slit-lamp examination after two years of scleral lens + autologous serum usage, revealing a persistently fully transparent and epithelized corneal graft.

In May 2017, the patient developed de novo refractory central neurotrophic ulcers in OU, with 5 mm diameter, approximately. Despite hourly preservative-free topical lubricants, topical preservative-free ofloxacin and hydrocortisone sodium phosphate four times per day, oral low-dose doxycycline, and oral acyclovir (initially in a therapeutic dose, posteriorly reduced to a prophylactic dose to account for a possible herpetic eye disease), no improvement was registered. By September 2017, the patient initiated hourly AS eyedrops while maintaining topical preservative-free ofloxacin and hydrocortisone sodium phosphate four times per day. Even though steroids may limit re-epithelization and promote secondary infections, the hydrocortisone sodium phosphate eye drops were important to limit the stromal inflammatory process, thus minimizing the risk of corneal melting and the dimensions of a future corneal leucoma. Furthermore, the topical preservative-free ofloxacin was effective in preventing secondary infection. In our institution, the serum is created by collecting venous blood samples from the patient, which are then submitted to a process of clotting, centrifuging, and diluting with sterile saline on the same day. This process is carried out by our hospital pharmacy. The serum is then stored in the freezer at minus 20 degrees Celsius. When it is ready to be released, the serum is unfrozen and divided into multiple sterile eye drop bottles. Once opened, the patient is instructed to keep these drops in their home refrigerator at 4 degrees Celsius. The patient had already completed a three-month period of oral low-dose doxycycline but was still receiving oral acyclovir in a prophylactic dose. By March 2018, the ulcer in her right eye (OD) improved to a smaller ulcer with 2.5mm diameter, approximately, while her OS presented complete graft re-epithelialization. In May 2018, her OD neurotrophic ulcer complicated with fungal and subsequent bacterial secondary infection. Eventually, a therapeutic penetrant keratoplasty was required for her OD, which was performed in October 2018. Figure 1 illustrates the evolution of the patient's OD, with an initial extensive central neurotrophic ulcer with stromal melting and haze (1A), before the OD penetrating keratoplasty (September 2018). Subsequently, her OD corneal graft developed a de novo 6x6mm central persistent epithelial defect unresponsive to all traditional therapeutic strategies and even new regenerating agents, such as Cacicol® (RGTA®; Laboratoires Théa, Clermont-Ferrand, France), which contains a polymer that mimics heparan sulfates bound to extracellular matrix proteins of the corneal stroma and epithelium, avoiding its proteolysis [8]. After months of unsuccessful treatment, we tried a new therapeutic option: a gas-permeable mini-scleral contact lens (Zenlens® toric, prolate, with a 16.00 mm diameter, 4950 micron sagittal depth, 7.10 mm base curve, advanced peripheral system of horizontal flat +3 and vertical steep -3, and a spherical power of -4.50D; Bausch & Lomb Incorporated, Ontario, Canada) in combination with AS eyedrops. The lens was worn continuously for 48h, being removed only for cleaning and replacing the fluid reservoir with AS. AS eyedrops were also applied hourly. After two weeks of this treatment regimen, the corneal epithelium eventually started to regenerate, and four weeks later, the cornea was completely re-epithelized. The continuous usage was then reduced to a 24h period. To date, there are no signs of recurrence of the corneal epithelial defect/ulcer. Figures 1B and 1C further depict the transparent and fully re-epithelized OD corneal graft after four weeks of scleral lens and AS eyedrops usage. After two years of this treatment regimen (with a 24h mini-scleral contact lens continuous usage period, AS fluid reservoir, and hourly AS eyedrops, as well as topical preservative-free ofloxacin gel and hydrocortisone sodium phosphate eyedrops twice daily), the OD corneal graft remained fully transparent and epithelized, with only a slight corneal stromal haze in the area of the previous ulcer, as Figure 1D lastly illustrates. This image was captured in February 2021.

**Figure 2:**
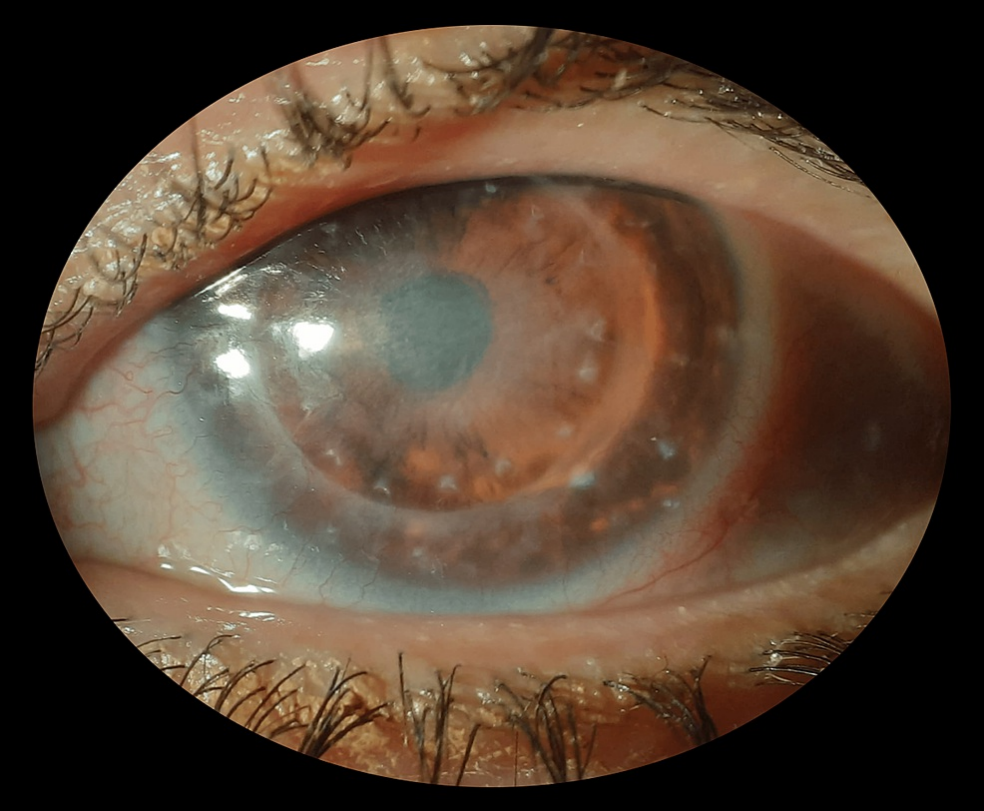
February 2021: OS slit-lamp photography with a completely epithelized cornea and a central leucoma

During the same period, the patient's OS was continuously treated with hourly AS eyedrops, as well as preservative-free hydrocortisone sodium phosphate eyedrops twice daily, remaining completely epithelized, despite a central leucoma from the previous ulcer. This is illustrated in Figure 2. 

**Figure 3:**
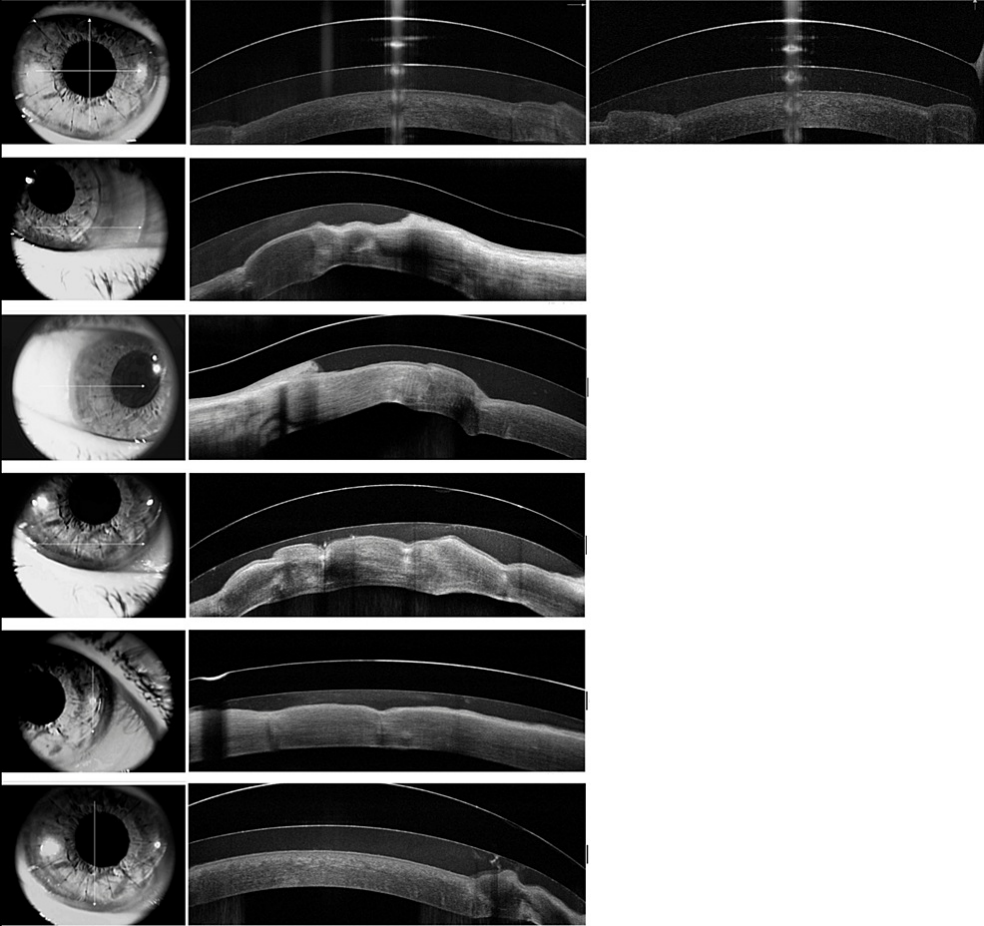
February 2021: OD anterior segment OCT demonstrating a regularly epithelized cornea with a gas-permeable mini scleral contact lens OCT - optical coherence tomography

Finally, Figure 3 corresponds to an anterior segment optical coherence tomography acquired with the Optovue AvantiTM optical coherence tomography imaging platform (Optovue, Fremont, CA), which demonstrates the OD regularly epithelized cornea with the gas-permeable mini-scleral contact lens, appropriately fitted to the patient's cornea. These images were also acquired in February 2021.

## Discussion

The treatment of NK remains a significant challenge, associated with elevated costs and a negative impact on the patients' vision-related quality of life (QoL) [9]. In a recent study by Bian et al., the authors determined that common procedures for NK management included the use of amniotic membrane (29.90%), punctal plugs (29.65%), and bandage contact lenses (BCL) (22.67%), as well as topical lubricants, which are used almost universally in these cases. Furthermore, in their study, older age, male sex, Black race, Hispanic or Latino ethnicity, unilateral involvement, concomitant diagnosis of diabetes, corneal transplant, or herpetic keratitis were significantly associated with worse final visual acuity (VA) [6]. Furthermore, Roumeau et al. systematically reviewed the efficacy of 5 treatment classes for NK in 20 studies that included 571 patients [7]. In their study, the percentage of patients with complete corneal healing did not differ between NGF eyedrops (75%; 95% CI 46 to 104%), AS eyedrops (92%; 95% CI 86 to 98%), corneal neurotization (99%; 95% CI 95 to 103%) and AMT (86%; 95% CI 78 to 94%) (p>0.05). All these specific treatments had a better percentage of complete epithelial healing (p<0.001) than isolated lubricants (23%; 95% CI 14 to 32%). Nevertheless, the time to complete healing was 24.2 days (5.4 to 43.1) with NGF eyedrops, 27.6 days (15.2 to 40.0) with AS eyedrops, 117 days (28.8 to 205.2) with corneal neurotization, and 16.4 days (11.1 to 21.7) with AMT and only NGF and AMT significantly improved VA. This study demonstrated that all these specific treatment modalities are valid for the management of NK while implying that NGF and AMT may lead to faster complete corneal epithelial healing and a more significant VA improvement [7]. Nonetheless, the authors acknowledge that further comparative clinical trials are still required to confirm the medical benefits of these expensive therapies and establish clinical guidelines for the management of NK [7].

This case highlights the potential value of continuous wear of a gas-permeable scleral contact lens combined with AS eyedrops as a novel and promising alternative approach to conventional treatments for refractory neurotrophic persistent defects. Thus, this treatment regimen combines the healing properties of the AS with the protective features of the scleral contact lens, which, being gas-permeable and adequately fitted to the patient's cornea, allows for appropriate oxygenation and nutrition of the cornea. By retaining the AS in contact with the patient's cornea for an extended period of time, the healing properties of the AS are intensified, and the cornea remains hydrated for a longer period of time. Even though multiple case reports and even prospective studies of persistent corneal epithelial defects successfully treated with a combined regimen of BCL and AS have already been published in the literature [10-13], our case is unique since it includes the fitting of a scleral contact lens, adjusted to an irregular corneal format, which is inevitable after a neurotrophic ulcer developed in an eye already submitted to a penetrating keratoplasty. Thus, this solution might be more appropriate for corneas with irregular formats since it will allow for a more stable lens fitting with better corneal lubrification and stabilization of the cornea growth-factor-rich microenvironment. Naturally, there are risks/complications associated with this treatment regimen, mainly related to the scleral lens, such as: (1) accumulation of debris in the postlens AS film reservoir during scleral lens wear, which can occur in up to 30% of patients who use scleral gas-permeable contact lenses [14], and causes central blurry vision, but can corrected with removal and reapplication of the scleral lens; (2) scleral misalignment, with or without conjunctival prolapse or compression/impingement, which can be corrected by adjusting the lens diameter or experimenting a new lens design, which can now be done with the aid of scleral topographers, such as the such as the sMap3D (Visionary Optics, Front Royal, Virginia), the Eye Surface Profiler (Eaglet Eye, Austin, Texas) and even scleral tomographers, such as the Pentacam (Oculus, Wetzlar , Germany) [15]; (3) complications which are shared by the multiple types of contact lens, such as mechanical and hypoxic keratitis (minimized by the gas-permeability and adequate fit), hypersensitivity keratitis with sterile marginal corneal infiltrates (hypersensitivity response to chemicals used in lens care), bacterial keratitis and contact lens-associated giant papillary conjunctivitis. Additionally, the process of obtaining AS eyedrops presents high costs, and its success in these cases relies on frequent application by the patient. Nonetheless, these complications can be detected early in most cases and managed with appropriate topical treatment with antibiotics and/or anti-inflammatory agents, and motivated patients will adhere to this treatment, especially when other conventional treatments have already failed, and there is significant visual impairment and ocular surface complaints. Furthermore, in this case, we opted against using an amniotic membrane since the dimensions of the ulcer (6x6mm, circular and central in the visual axis) limited its efficacy, as previously reported in the literature [16]. The patient's extreme dry eye also required continuous lubrification, which was optimized with the AS eyedrops, and continuous use of the mini-scleral contact lens, which allowed continuous lubrification and the creation of a growth-factor-rich corneal microenvironment.

Since 2015, plasma rich in growth factor (PRGF) eye drops have emerged as a non-inferior alternative to AS eye drops. Some studies have even reported higher levels of some growth factors (such as platelet-derived growth factor-AB, vascular endothelial growth factor, epidermal growth factor, fibroblast growth factor, and transforming growth factor-beta1) in PRGF eye drops when compared with AS eye drops. Furthermore, PRGF eye drops may be superior in preventing and inhibiting TGF-β1-induced myofibroblast differentiation in the corneal stroma, thus minimizing corneal scar formation when compared to AS eye drops [17]. Nonetheless, in our institution, the protocol for PRGF eye drops preparation is not yet established. Thus, we have not been able to experiment with this valuable alternative in our patients yet and compare its results with the conventional AS eye drops in patients with persistent corneal epithelial defects.

## Conclusions

In our opinion, the benefits of the treatment regimen in this case far outweigh the potential complications, combining two extremely useful and frequently employed treatments for NK: AS eyedrops and scleral contact lenses. The scleral contact lens might be more appropriate for corneas with irregular formats (frequently found in eyes with refractory corneal epithelial defects) than regular BCL, since it will allow for a more stable lens fitting with better corneal lubrification and stabilization of the cornea growth factor rich microenvironment provided by the AS. Naturally, our clinical experience with this empirical approach remains limited and further investigation is required before universal clinical validation.
